# A Case of Adult Pancreatoblastoma With Novel *APC* Mutation and Genetic Heterogeneity

**DOI:** 10.3389/fonc.2021.725290

**Published:** 2021-08-27

**Authors:** Yamato Suemitsu, Yusuke Ono, Yusuke Mizukami, Juanjuan Ye, Keiko Yamakawa, Takeshi Takamoto, Yuko Nakano-Narusawa, Yuri Mukai, Manabu Takamatsu, Atsuko Nakazawa, Mari Mino-Kenudson, Toshio Kumasaka, Yoko Matsuda

**Affiliations:** ^1^Department of Pathology, Japanese Red Cross Medical Center, Shibuya, Japan; ^2^Cancer Genetics, Department of Medicine, Asahikawa Medical University, Asahikawa, Japan; ^3^Institute of Biomedical Research, Sapporo-Higashi Tokushukai Hospital, Sapporo, Japan; ^4^Oncology Pathology, Department of Pathology and Host-Defense, Faculty of Medicine, Kagawa University, Kita-gun, Japan; ^5^Department of Hepatobiliary and Pancreatic Surgery, National Cancer Center Hospital, Chuo-ku, Japan; ^6^Clinicopathology Center, Cancer Institute Hospital of JFCR, Koto-ku, Japan; ^7^Division of Pathology, Saitama Children’s Medical Center, Saitama, Japan; ^8^Department of Pathology, Massachusetts General Hospital, Boston, MA, United States

**Keywords:** pancraetoblastoma, basophilic cells, clear cells, APC, cartilaginous differentiation, solid pseudopaillary neoplasm of the pancreas, pancreatic neuroendocrine tumor (PanNET)

## Abstract

**Background:**

Pancreatoblastoma is a rare malignant epithelial neoplasm of the pancreas that mainly occurs in children and involves abnormalities in the WNT/β-catenin pathway, such as *CTNNB1* mutation. However, the molecular abnormalities in adult pancreatoblastoma are not well known.

**Case Presentation:**

An elderly man, who underwent elective distal pancreatectomy and splenectomy, was referred to our hospital with a mass in the tail of the pancreas. Histologically, the lesion revealed proliferation of clear, basophilic, and cartilaginous tumor cells with lymphatic metastasis. Each of the morphologically distinct tumor components showed different immunohistochemical patterns, indicating heterogeneous differentiation, including epithelial (both acinar and ductal), mesenchymal, and neuroendocrine differentiation. All tumor components showed nuclear expression of β-catenin and cyclin D1. Per next-generation sequencing (NGS), the clear and basophilic tumor cells shared mutations in *APC, GRM8*, *LAMP1*, and *AKA9*. Among the mutations, *APC*, c.1816_1817insA showed the highest frequency in both cell types, indicating that *APC* mutation was a driver mutation of the tumor. A diagnosis of PB was rendered.

**Summary:**

In conclusion, the clear and basophilic cells of the tumor were supposedly derived from the same clone and subsequently acquired additional mutations. This is the first report of clonal evolution in pancreatoblastoma.

## Introduction

Pancreatoblastoma (PB) is a rare malignant epithelial neoplasm of the pancreas that demonstrates multiple patterns of differentiation. “Infantile pancreatic carcinoma” is a term previously used to describe this condition. Horie et al. proposed the term “pancreatoblastoma” (1) based on the histological resemblance of the tumors to fetal pancreatic tissues at a gestational age of 7 weeks. PB is commonly seen in the pediatric population, where it accounts for approximately 25% of pancreatic neoplasms. On the other hand, it is very rare in the adult population (2), and making a preoperative diagnosis of PB is challenging because of the limited roles of tumor markers and cellular heterogeneity of the tumors, the latter of which makes fine-needle aspiration (FNA) cytology and histology indecisive, considering the limitations of sampling (3). After diagnosis, PB in adults, with a median survival time of 18.5 months is associated with poorer prognosis than in children despite aggressive treatments (2). Deletion of 11q is the most common genetic abnormality reported in PB.

Approximately 50%–80% of PB cases show abnormalities in the WNT/β-catenin pathway, and among them, *CTNNB1* mutations are most frequently seen. Nuclear expression of β-catenin is observed by immunohistochemical analysis of the PB. *KRAS*, *TP53*, and *SMAD4*, all of which are frequently mutated in pancreatic ductal adenocarcinomas, are usually unaffected in PB. To date, approximately 40 cases of patients over 18 years of age have been reported, and due to its rarity and the limited number of reports, the pathogenesis of adult PB remains unclear. Here, we report a case of PB in an elderly patient who received a preliminary diagnosis of combined solid pseudopapillary neoplasm and neuroendocrine tumor; however, the diagnosis was later determined to be PB, based on the results of NGS. We describe the molecular profile of adult PB along with a review of previous literature.

## Clinical Course

**Figure 1:**
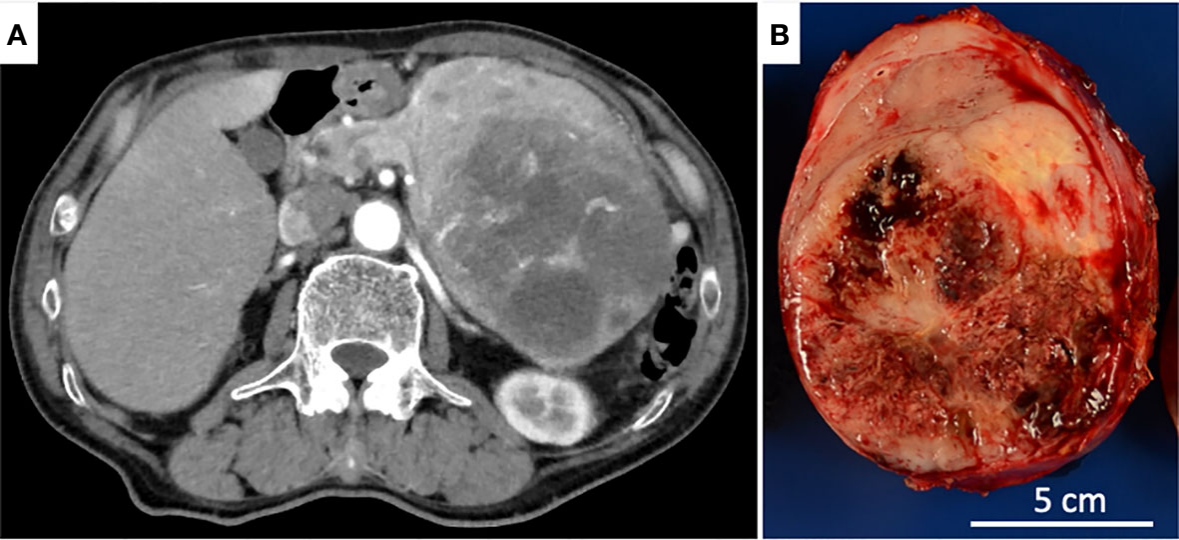
**(A)** Axial CT revealed an expansile, solid tumor largely degenerated with necrosis in the dorsal part. **(B)** The cut surface of the pancreatic tail tumor showed a well-circumscribed, lobulated, solid tumor with a large hemorrhagic, necrotic area.

A 74-year-old male patient, without any relevant past or familial medical history, presented with anorexia and unintentional weight loss over two weeks. On examination, a firm non-tender intra-abdominal mass was palpated in the upper right quadrant. Blood tests showed nonspecific inflammatory reaction, and none of the tumor markers were elevated: carcinoembryonic antigen, 1.9 ng/mL; carbohydrate antigen, 19-9 10 U/mL. Computed tomography (CT) with contrast enhancement revealed a solid, well-demarcated lesion in the tail of the pancreas with partial necrotic degeneration that was up to 130 mm in size. Distant metastasis was not observed (**Figure 1A**). Endoscopic ultrasound-guided FNA was performed, leading to a histological diagnosis of a neuroendocrine tumor. The patient underwent elective open distal pancreatectomy and splenectomy.

## Pathological Findings

The tumor was grossly round and encapsulated, measuring 14.0 x 10.3 x 8.1 cm, and the cut surfaces showed a well-circumscribed, lobulated, solid tumor largely degenerated with hemorrhage and necrosis. The non-neoplastic pancreatic tissue was hardly noticeable (**Figure 1B**).

**Figure 2:**
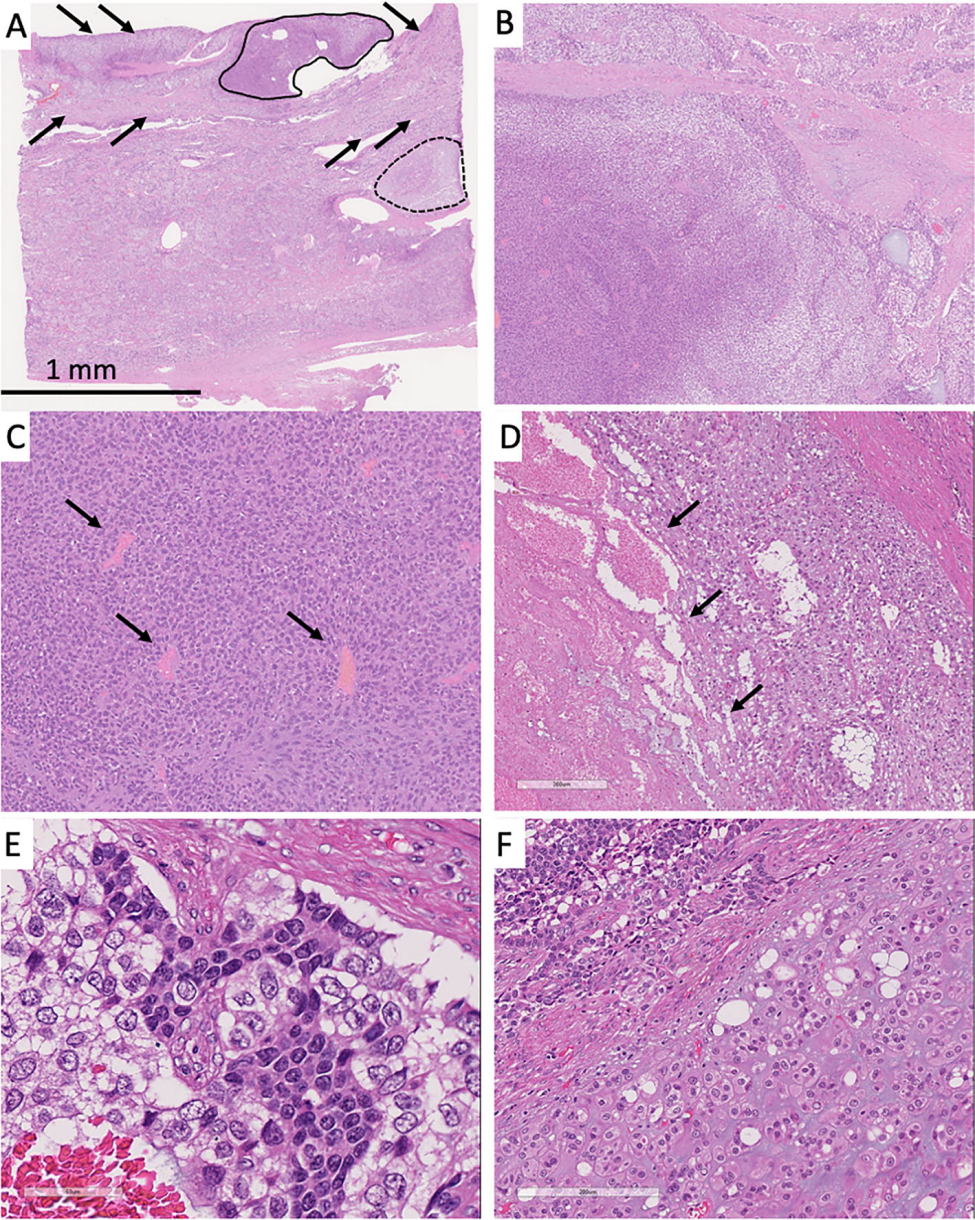
**(A)** Solid tumor had the three characteristic cell components: conventional neuroendocrine tumor lesion (solid line), clear cells (arrows), and tumor cells with cartilaginous differentiation (dotted line). Most of the tumor showed mixture of the basophilic and clear cells. **(B)** The left part predominantly showed basophilic tumor cells and the right part clear cells. **(C)** Basophilic cells showed rosette patterns (arrows). **(D)** Clear cells showed necrosis (arrows). **(E)** Most of the tumor showed sheet-like appearance and mixture of basophilic and clear cells. Basophilic cells showed small round nuclei. Clear cells showed small to medium-size clear nuclei with thickened nuclear membrane. **(F)** The lower part showed cartilaginous differentiation, and the upper part was composed of basophilic and clear cells.

Histologically, three distinct cell components were observed: basophilic cells (solid line, **Figure 2A**), clear cells (arrows, **Figure 2A**), and cartilaginous differentiation (dotted line, **Figure 2A**). The areas of the basophilic and clear cells were closely intermixed (**Figure 2B**). The basophilic cells showed glandular or rosette-like structures with inconspicuous nucleoli (**Figure 2C**). The clear cells showed a sheet-like growth pattern with focal necrosis and small to medium nucleoli with pale chromatin, thickened nuclear membrane, and vacuolated cytoplasm (**Figure 2D**). Most of the tumors showed a mixture of these two cell components without clear boundaries (**Figure 2E**). Partly, there was a collection of tumor cells that showed cartilaginous differentiation with round small nucleoli and chondroid stroma (**Figure 2F**). The tumor was encapsulated and clearly demarcated from the remaining non-neoplastic pancreatic tissue. However, it invaded the peripancreatic soft tissue anteriorly. Three peripancreatic lymph nodes showed metastasis as follows: two were composed of both basophilic and clear cells, and one was composed solely of basophilic cells.

**Figure 3:**
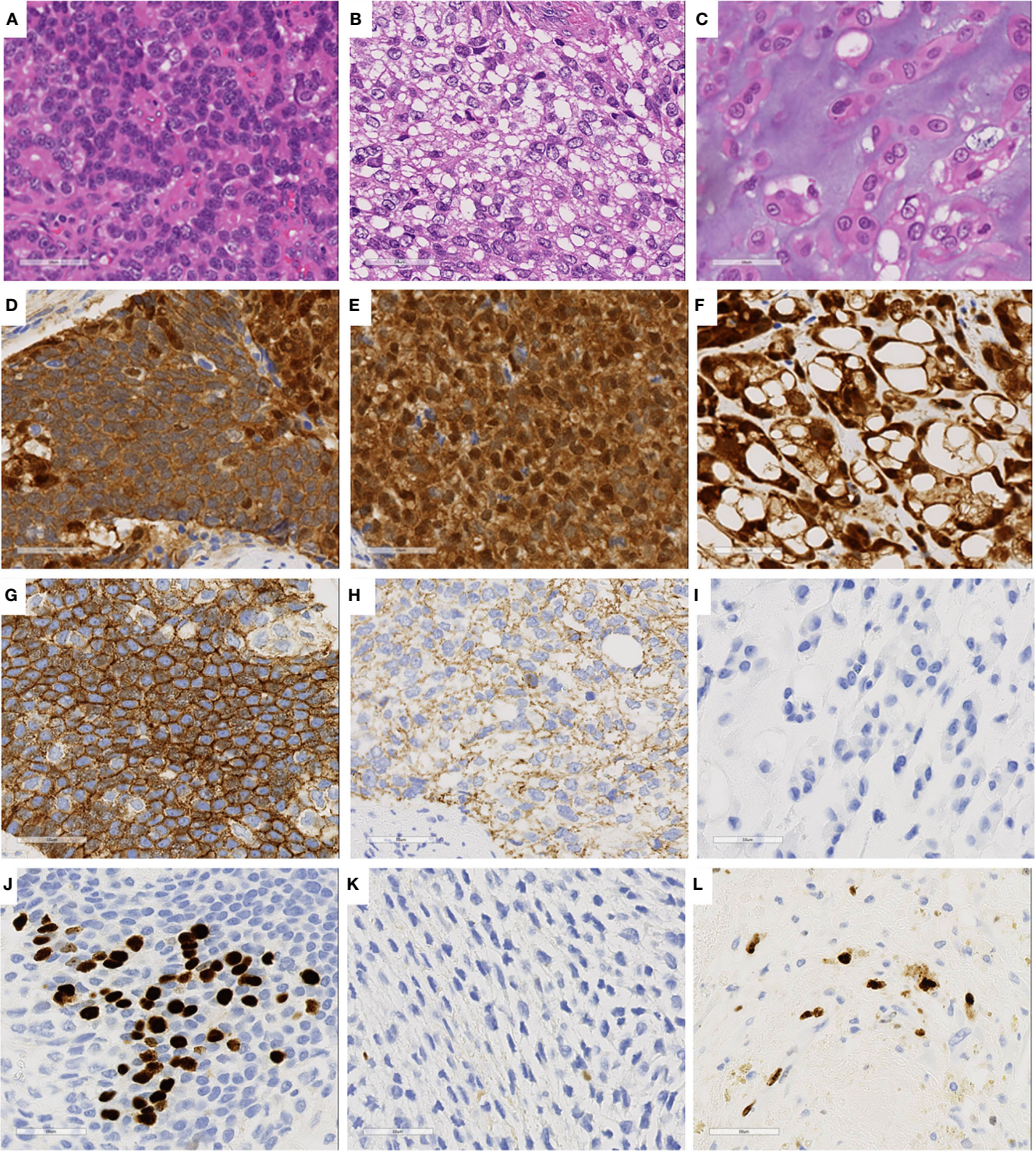
Immunohistochemical staining of three characteristics cell components. **(A, D, G, J)** Basophilic cell lesion. **(B, E, H, K)** Clear cell lesion. **(C, F, I, L)** Cartilage-like component. **(D–F)**, β-catenin; **(G–I)** E-cadherin; **(J–L)** Ki67.

Immunohistochemical analysis of the three cell components showed different expression patterns (**Table 1**, **Supplementary Table 1,** and **Supplementary Information**). The basophilic cells (**Figure 3A**) showed weak nuclear expression of β-catenin (**Figure 3D**) and strong circumferential membrane E-cadherin staining (**Figure 3G**). The clear cells (**Figure 3B**) exhibited strong nuclear expression of β-catenin (**Figure 3E**) and decreased membranous staining of E-cadherin (**Figure 3H**). In the cells with cartilaginous differentiation (**Figure 3C**), nuclear β-catenin accumulation was observed (**Figure 3F**), but no membranous staining of E-cadherin was observed (**Figure 3I**). The Ki-67 (MIB-1) labeling index of the basophilic cells, clear cells, and cartilage-like cells was approximately 30%, less than 1%, and 20%, respectively (**Figures 3J–L**).

**Table 1:** Immunohistochemical staining.

	Basophilic cells	Clear cells	Cartilaginous differentiation
Ki67 (%)	30	<1	20
bcl-10	+	+, weak	+, weak
Alfa antitrypsin	–	+	+
Chymotrypsin	–	+	+
Amylase	–	–	–
Trypsin	–	–	–
Synaptophysin	+	+, weak	–
Chromogranin A	+	–	–
CD56	+	+	+
NSE	+	+	+
SOX9	+, weak	+, weak	+
CEA	–	–	–
CA19-9	–	–	–
AE1/AE3	+	+	–
Cytokeratin 19	–	–	–
Cytokeratin 7	–	–	–
34betaE12	–	–	–
p40	–	–	–
β-catenin (nuclear)	+, weak	+	+
E-cadherin	+	+, weak	–
Vimentin	–	+	+
Cyclin D1	+	+	+
LEF1	+	+	+
S100	–	+	+
Estrogen recepter	–	–	–
Progesterone recepter	–	+	–
CD10	–	+	+, weak

Bcl-10, an acinar cell marker, was diffusely expressed in basophilic cells, while its expression was weaker in the other two cell lines (**Supplementary Figures 1A–C**). Alfa antitrypsin and chymotrypsin were positive in clear cells and those with cartilaginous differentiation but negative in basophilic cells. The basophilic cells showed stronger positivity for synaptophysin than the other two cells that exhibited weak or no expression (**Supplementary Figures 1D–F**). Chromogranin A showed the same results as synaptophysin. All lesions were positive for CD56 and NSE. SOX9, a pancreatic ductal cell marker, was highly expressed in the cartilaginous component, but only weakly expressed in other tissues (**Supplementary Figures 1G–I**). The basophilic, clear, and cartilage-like cells showed negative, weak or strong vimentin expression (**Supplementary Figures 1J–L**), respectively, in contrast to the E-cadherin expression patterns. LEF1, a marker for solid pseudopapillary neoplasms, was consistently expressed in all three components (**Supplementary Figures 1M–O**). These immunohistochemical results, in conjunction with the cellular morphological appearances, indicated multilineage differentiation of the tumor cells and a possible diagnosis of PB. However, we were not able to find any squamous morule, which is usually confirmed by 34betaE12 and p40 staining, and a tentative histological diagnosis of combined solid pseudopapillary neoplasm and neuroendocrine tumor (G3) was made.

## Gene Mutation Analysis

We extracted DNA from the three tumor cell components and analyzed mutations in 409 cancer-related genes (**Supplementary Table 2** and **Supplementary Information**), although we were not able to obtain a sufficient amount of DNA from the cells with cartilaginous differentiation. DNA extracted from the spleen was used as a control. The specific mutations in *APC, GRM8*, *LAMP1, and AKA9* were shared by basophilic cells and clear cells (**Table 2**). Of these, the *APC* c.1816_1817insA mutation showed the highest frequency in both lesions (71% and 81%, respectively), suggesting that the *APC* mutation was a driver mutation in the present case. In addition, *ROS1* and *SDHA* mutations were present in the basophilic cells, and *KMT2C, DDR2, FANCD2*, and *FGFR2* mutations in clear cells. The results indicated that the two components were derived from the same clone and acquired additional mutations. On the other hand, the genes commonly altered in other types of pancreatic tumors, such as *KRAS, TP53, SMAD4, CCND1*, and *ATRX*, were unaffected in the present case, and neither did CTNNB1.

**Table 2:** Gene mutations determined by Comprehensive Cancer Panel.

Chromosome	Gene	Mutation	Protein	Frequency of gene mutations (%)	
				Basophilic cells	Clear cells
chr5	*APC*	c.1816_1817insA	p.Ile606AsnfsTer28	71.0	81.3
chr7	*GRM8*	c.2722A>G	p.Ile908Val	30.8	27.0
chr13	*LAMP1*	c.626C>A	p.Ser209Ter	28.1	31.6
chr7	*AKAP9*	c.1389G>T	p.Met463Ile	12.0	34.5
chr6	*ROS1*	c.2187G>A	p.Trp729Ter	16.6	N.D.
chr5	*SDHA*	c.1073G>A	p.Gly358Asp	14.6	N.D.
chr7	*KMT2C*	c.9235C>T	p.Arg3079Ter	N.D.	8.0
chr1	*DDR2*	c.1293+4A>T	p.?	N.D.	6.3
chr3	*FANCD2*	c.1214A>G	p.Asn405Ser	N.D.	4.6
chr10	*FGFR2*	c.2180A>G	p.Asn727Ser	N.D.	2.7

chr, choromosome; N.D., not detected.

## Final Diagnosis and Patient Follow-Up

The differential diagnosis of the present case included neuroendocrine tumor, solid pseudopapillary neoplasm, pancreatic ductal carcinoma, acinar cell carcinoma, PB, and a mixed tumor. We compared the gene mutations in the present case with those in the COSMIC database (**Supplementary Table 3**); however, the present case did not show any of the common mutations observed in neuroendocrine tumors (*MEN1, ATRX, and DAXX*), solid pseudopapillary neoplasms (*CTNNB1 and KDM6A*), ductal carcinomas (*KRAS, TP53, SMAD4, and CDKN2A*), acinar cell carcinomas (*SMAD4, TP53*, and *CTNNB1*), or PB (*CTNNB1*). Although the absence of squamoid nests and the presence of massive necrosis were not typical, we considered the multiple patterns of differentiation, including acinar, neuroendocrine, ductal, and cartilaginous components most consistent with PB. In the literature, *APC* mutations have been reported in PB (**Supplementary Table 4**), which supports the diagnosis of PB in this case.

The patient has been free from recurrence for 36 months without adjuvant therapy, despite the observed aggressive histological features including a high Ki-67-labeling index, necrosis, peripancreatic fat tissue invasion, and several lymph node metastases.

## Comments

APC is a negative regulator that controls β-catenin concentration and interacts with E-cadherin. Loss of APC protein leads to accumulation of β-catenin in the nuclei; thus, it is associated with an increase in transcription of Wnt-responsive genes (4). In the present case, the tumor cells showed nuclear expression of β-catenin and an *APC* mutation at high mutant allele frequencies, indicating the *APC* mutation as the driver mutation. *APC*, c.1816_1817insA, has been reported in familial adenomatous polyposis (5), but not in pancreatic tumors, including PB. Previous case reports showed that there were two cases of PB with *APC* mutations (6, 7) (**Supplementary Table 4**). Furthermore, the three cell components of the tumor in the present case showed different expression patterns of β-catenin, E-cadherin, and vimentin, suggesting heterogeneous WNT pathway alterations in the tumor cells.

The basophilic and clear cells shared four genetic mutations, and each component showed two and four additional genetic mutations, respectively. This suggests that these cells were derived from the same clone and then acquired different genetic alterations, which supposedly resulted in distinct histological types. This is the first report to reveal the clonal evolution of PB. Mutations in *KMT2C, DDR2, FANCD2*, and *FGFR2* were detected only in clear cells. Among them, FGFR2 phosphorylates specific tyrosine residues that mediate interaction with cytosolic adaptor proteins and the RAS-MAPK and PI3K-AKT intracellular signaling pathways, which regulate the binding of GSK-3β and β-catenin (8); thus, the mutations detected in the clear cell component might explain the varied expression of β-catenin, E-cadherin, and vimentin in the present case. LEF1, a specific diagnostic marker for solid pseudopapillary neoplasm (9), is a transcription factor also involved in WNT pathway. It recruits the coactivator β-catenin to enhancer elements of the genes and activates transcription of cyclin D1, c-jun, and c-myc (10), and its expression was observed in all the cell components.

The clear cells were morphologically similar to the cells seen in subtypes of neuroendocrine tumor: clear cell type (11, 12), spindle cell type (13), and neuroendocrine tumor with sarcomatoid differentiation (14). Immunohistochemical staining showed that the clear cells were positive for chromogranin A, synaptophysin, CD56, NSE, and AE1/AE3, indicating neuroendocrine differentiation of the cells. Furthermore, they showed a mutation in *KMT2C*, a gene that regulates chromatin remodeling (15). *KMT2C* mutations have been reported in 11% of neuroendocrine tumors (**Supplemental Table 3**).

Immunohistochemistry of the basophilic cells morphologically resembled acinar cell carcinoma and showed bcl-10 expression; however, they were not reactive to other acinar cell markers (such as alpha-antitrypsin, chymotrypsin, amylase, and trypsin), which made the differential diagnosis less likely. Cartilaginous differentiation of pancreatic tumors is generally rare, and we found a report of intraductal tubulopapillary neoplasm of the pancreas with stromal osseous and cartilaginous metaplasia (16). A portion of the tumor in the present case contained small round tumor cells with chondroid stroma. SOX9, which is expressed by such distinct tumor cells, is known to be a useful immunohistochemistry marker for detecting the ductal lineage of pancreatic tumors (17); moreover, it has been reported as an important transcription factor for chondrocyte differentiation (18). The strong expression of SOX9 in the selected tumor cells in the present case is thus related to their cartilaginous differentiation.

In conclusion, the present case represents an adult-onset PB with unusual morphological features, and the diagnosis was supported by immunohistochemistry and mutation analyses of various types of tumor cells. Furthermore, the molecular profiling of the tumor indicated differentiation from a single clone to multiple cell lineages along with the acquisition of distinct sets of genetic alterations. Thus, it is essential to recognize the possible morphological and genetic aspects of PB to make an accurate diagnosis.

## Data Availability Statement

The datasets presented in this study can be found in online repositories. The names of the repository/repositories and accession number(s) can be found in the article/**Supplementary Material**.

## Ethics Statement

The studies involving human participants were reviewed and approved by Japanese Red Cross Medical Center Ethics Committee. The patients/participants provided their written informed consent to participate in this study. Written informed consent was obtained from the individual(s) for the publication of any potentially identifiable images or data included in this article.

## Author Contributions

YS, MM-K, and YM wrote the manuscript. YS, YN-N, JY, AN, MM-K, TK, and YM was in charge of pathological diagnosis and histological study. YO, YMi, YMu and KY carried out genetic analysis. YO and YMi was in charge of genetic sequencing. TT and MT collected clinical data. All authors contributed to the article and approved the submitted version.

## Conflict of Interest

The authors declare that the research was conducted in the absence of any commercial or financial relationships that could be construed as a potential conflict of interest.

## Publisher’s Note

All claims expressed in this article are solely those of the authors and do not necessarily represent those of their affiliated organizations, or those of the publisher, the editors and the reviewers. Any product that may be evaluated in this article, or claim that may be made by its manufacturer, is not guaranteed or endorsed by the publisher.
