# Supplementary material for: A Case of Adult Pancreatoblastoma With Novel APC Mutation and Genetic Heterogeneity
Source: Front Oncol. 2021 Aug 27;11:725290. doi: 10.3389/fonc.2021.725290 (PMC8432961; doi:10.3389/fonc.2021.725290)
Supplement: Supplementary file 1 [file DataSheet_1.docx]

**Supplemental information**

***Pathological analysis***

Pancreas tissues were fixed in 10% buffered formalin and then subjected to standard tissue processing and paraffin embedding. The tissues were sliced serially into sections 3 μm thick for hematoxylin and eosin (H&E) and immunohistochemical staining. Bound antibodies were detected using diaminobenzidine tetrahydrochloride as the substrate. The sections were then counterstained with Mayer's hematoxylin. Negative control tissue sections were prepared by omitting incubation with the primary antibody.

***Targeted amplicon sequencing***

We analyzed the sequences of 409 cancer related genes using Ion Ampliseq Comprehensive Cancer Panel (Thermo Fisher Scientific). All genes ware listed on Supplemental Table 1. The panel consisted of 16,000 amplicons divided into four primer pools totaling 1.75 Mb of DNA. DNA (20 ng) was amplified using this panel, and a sequencing library was prepared using Ion Ampliseq Library Kit Plus (Thermo Fisher Scientific). Sequencing and data analyses were performed using the Ion S5 GeneStudio system (Thermo Fisher Scientific) as previously described [19]. Sequenced reads were demultiplexed, quality-filtered, and aligned to the human reference genome (GRCh37) by Torrent Suite software package (ver. 5.12.2; Thermo Fisher Scientific). The mapping module (Torrent Mapping Alignment Program) is a sequence alignment software program optimized specifically for Ion Torrent data and includes several mapping algorithms, each with its own best application. Mapping was completed with its default parameter values. Variants were identified using the Variant Caller plugin (ver. 5.12.0.4; Thermo Fisher Scientific) included in the Torrent Suite Package, which is optimized to exploit the underlying flow signal information. Variant calling analysis was completed using the somatic variant calling mode, which is optimized to detect low-frequency variants. The setting parameters were as follows: minimum allele frequency, 0.02; minimum coverage, 40. To identify somatic mutations, independent genotyping results for the tumor and normal samples were subtracted, and variants found in the normal samples were excluded from the molecular profiling. Allele variants of low coverage (9 or fewer) were excluded in this study, and putative false-negative variants were excluded by manually confirming the alignment by IGV software (version 2.3.59; <http://software.broadinstitute.org/software/igv/>). For variants containing novel exonic, nonsynonymous, and frameshift variants as well as intronic splice variants, the COSMIC (http://cancer.sanger.ac.uk/cosmic) and ClinVar databases (https://www.ncbi.nlm.nih.gov/clinvar/) were used for classification as either pathogenic or a variant of unknown significance [1].

**Reference**

1. Omori, Y. et al. Pathways of progression from intraductal papillary mucinous neoplasm to pancreatic ductal adenocarcinoma based on molecular features. Gastroenterology 156, 647-661 e642 (2019).
