# Supplementary figures and images for: A Case of Adult Pancreatoblastoma With Novel APC Mutation and Genetic Heterogeneity
Source: Front Oncol. 2021 Aug 27;11:725290. doi: 10.3389/fonc.2021.725290 (PMC8432961; doi:10.3389/fonc.2021.725290)

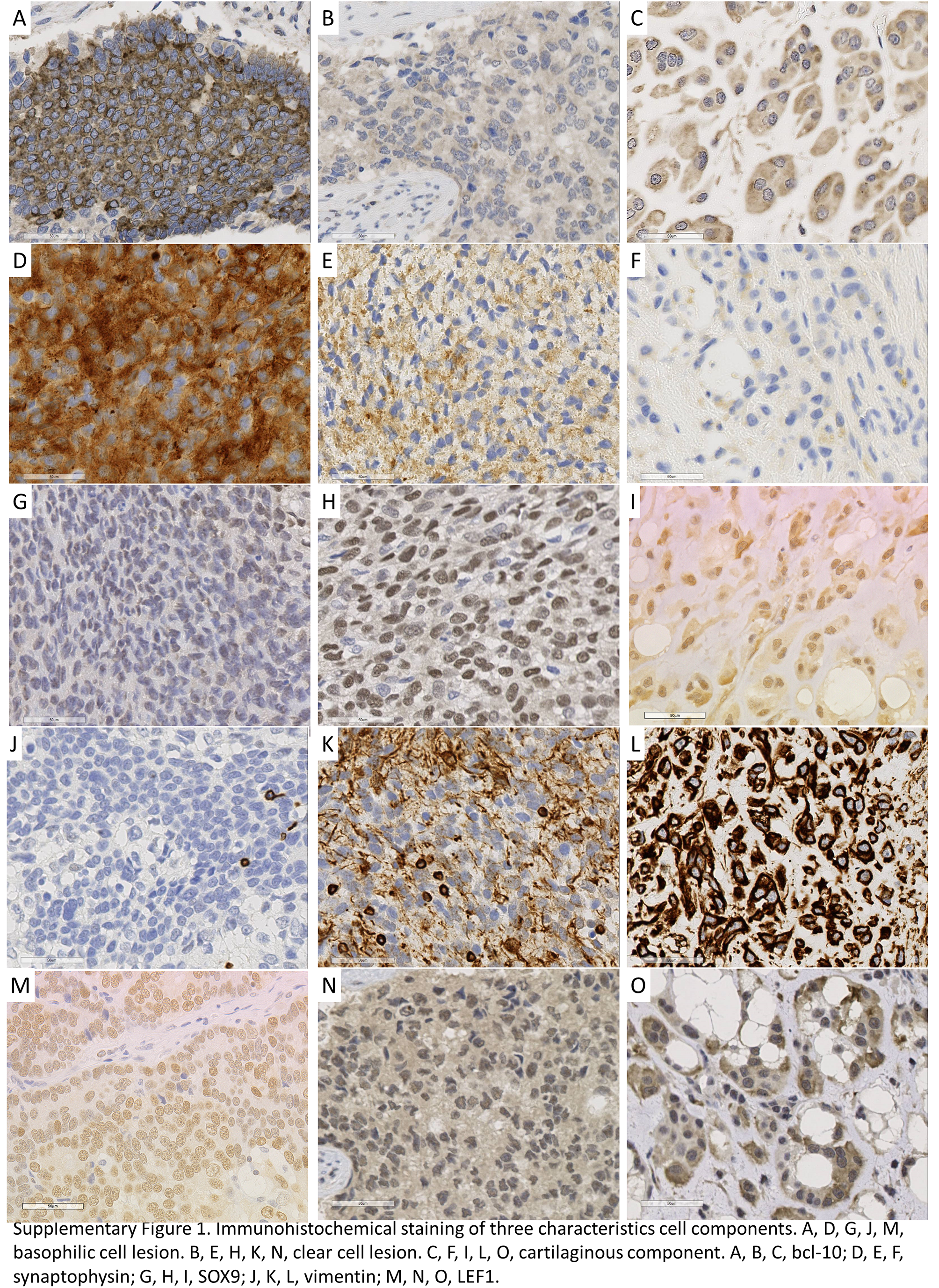

Supplement: Supplementary file 2 [file Image_1.tif]
