# Supplementary material for: A Case of Adult Pancreatoblastoma With Novel APC Mutation and Genetic Heterogeneity
Source: Front Oncol. 2021 Aug 27;11:725290. doi: 10.3389/fonc.2021.725290 (PMC8432961; doi:10.3389/fonc.2021.725290)
Supplement: Supplementary file 3 [file Table_1.doc]

| Supplementary Table 1. Antibodies for immunostaining | | |  |  |
| --- | --- | --- | --- | --- |
| Primary antibodies | Dilution | Antigen retrieval | Clone | Company |
| Ki67 | 1:100 | pH9 | MIB1 | Dako |
| bcl-10 | 1:50 | pH9 | 331.3 | Santa Cruz |
| Alfa antitrypsin | 1:3200 | - | - | Dako |
| Chymotrypsin | 1:200 | - | - | Thermo Fisher |
| Amylase | 1:150 | - | - | Cosmo Bio |
| Trypsin | 1:500 | - | - | Meridian |
| Synaptophysin | 1:200 | pH9 | 27G12 | Leica biosystems |
| Chromogranin A | 1:3000 | pH9 | - | Dako |
| CD56 | ready to use | pH9 | CD564 | Leica biosystems |
| NSE | ready to use | pH9 | 22C9 | Leica biosystems |
| SOX9 | 1:500 | pH6 | - | Atlas antibodies |
| CEA | 1:100 | - | - | Dako |
| CA19-9 | 1:180 | pH9 | 1116NS19.9 | Japan Tanner corp |
| AE1/AE3 | 1:300 | pH9 | AE1/AE3 | Leica biosystems |
| Cytokeratin 19 | 1:150 | proteinase K | RCK108 | Dako |
| Cytokeratin 7 | ready to use | pH9 | RN7 | Leica biosystems |
| 34betaE12 | ready to use | pH9 | 34βE12 | Leica biosystems |
| p40 | ready to use | pH9 | 738171 | Nichirei corp. |
| beta catenin (nuclear) | 1:1000 | pH9 | 14/Beta-Catenin | BD |
| E-cadherin | 1:100 | pH9 | 36B5 | Leica biosystems |
| Vimentin | 1:600 | pH6 | V9 | Dako |
| Cyclin D1 | ready to use | pH9 | EP12 | Leica biosystems |
| LEF1 | 1:200 | pH6 | - | Atlas antibodies |
| S100 | 1:1600 | pH6 | - | Dako |
| Estrogen receptor | ready to use | pH9 | 6F11 | Leica biosystems |
| Progesterone receptor | 1:600 | pH9 | NCL-L-PGR-312 | Leica biosystems |
| CD10 | ready to use | pH9 | 56C6 | Leica biosystems |

| Supplementary Table 2. 409 genes anazed in the present case | | | | | |  |  |  |
| --- | --- | --- | --- | --- | --- | --- | --- | --- |
| *ABL1* | *BTK* | *DDR2* | *FLT1* | *ITGA9* | *MLL* | *PALB2* | *RALGDS* | *TCF12* |
| *ABL2* | *BUB1B* | *DEK* | *FLT3* | *ITGB2* | *MLL2* | *PARP1* | *RARA* | *TCF3* |
| *ACVR2A* | *CARD11* | *DICER1* | *FLT4* | *ITGB3* | *MLL3* | *PAX3* | *RB1* | *TCF7L1* |
| *ADAMTS20* | *CASC5* | *DNMT3A* | *FN1* | *JAK1* | *MLLT10* | *PAX5* | *RECQL4* | *TCF7L2* |
| *AFF1* | *CBL* | *DPYD* | *FOXL2* | *JAK2* | *MMP2* | *PAX7* | *REL* | *TCL1A* |
| *AFF3* | *CCND1* | *DST* | *FOXO1* | *JAK3* | *MN1* | *PAX8* | *RET* | *TET1* |
| *AKAP9* | *CCND2* | *EGFR* | *FOXO3* | *JUN* | *MPL* | *PBRM1* | *RHOH* | *TET2* |
| *AKT1* | *CCNE1* | *EML4* | *FOXP1* | *KAT6A* | *MRE11A* | *PBX1* | *RNASEL* | *TFE3* |
| *AKT2* | *CD79A* | *EP300* | *FOXP4* | *KAT6B* | *MSH2* | *PDE4DIP* | *RNF2* | *TGFBR2* |
| *AKT3* | *CD79B* | *EP400* | *FZR1* | *KDM5C* | *MSH6* | *PDGFB* | *RNF213* | *TGM7* |
| *ALK* | *CDC73* | *EPHA3* | *G6PD* | *KDM6A* | *MTOR* | *PDGFRA* | *ROS1* | *THBS1* |
| *APC* | *CDH1* | *EPHA7* | *GATA1* | *KDR* | *MTR* | *PDGFRB* | *RPS6KA2* | *TIMP3* |
| *AR* | *CDH11* | *EPHB1* | *GATA2* | *KEAP1* | *MTRR* | *PER1* | *RRM1* | *TLR4* |
| *ARID1A* | *CDH2* | *EPHB4* | *GATA3* | *KIT* | *MUC1* | *PGAP3* | *RUNX1* | *TLX1* |
| *ARID2* | *CDH20* | *EPHB6* | *GDNF* | *KLF6* | *MUTYH* | *PHOX2B* | *RUNX1T1* | *TNFAIP3* |
| *ARNT* | *CDH5* | *ERBB2* | *GNA11* | *KRAS* | *MYB* | *PIK3C2B* | *SAMD9* | *TNFRSF14* |
| *ASXL1* | *CDK12* | *ERBB3* | *GNAQ* | *LAMP1* | *MYC* | *PIK3CA* | *SBDS* | *TNK2* |
| *ATF1* | *CDK4* | *ERBB4* | *GNAS* | *LCK* | *MYCL1* | *PIK3CB* | *SDHA* | *TOP1* |
| *ATM* | *CDK6* | *ERCC1* | *GPR124* | *LIFR* | *MYCN* | *PIK3CD* | *SDHB* | *TP53* |
| *ATR* | *CDK8* | *ERCC2* | *GRM8* | *LPHN3* | *MYD88* | *PIK3CG* | *SDHC* | *TPR* |
| *ATRX* | *CDKN2A* | *ERCC3* | *GUCY1A2* | *LPP* | *MYH11* | *PIK3R1* | *SDHD* | *TRIM24* |
| *AURKA* | *CDKN2B* | *ERCC4* | *HCAR1* | *LRP1B* | *MYH9* | *PIK3R2* | *SEPT9* | *TRIM33* |
| *AURKB* | *CDKN2C* | *ERCC5* | *HIF1A* | *LTF* | *NBN* | *PIM1* | *SETD2* | *TRIP11* |
| *AURKC* | *CEBPA* | *ERG* | *HLF* | *LTK* | *NCOA1* | *PKHD1* | *SF3B1* | *TRRAP* |
| *AXL* | *CHEK1* | *ESR1* | *HNF1A* | *MAF* | *NCOA2* | *PLAG1* | *SGK1* | *TSC1* |
| *BAI3* | *CHEK2* | *ETS1* | *HOOK3* | *MAFB* | *NCOA4* | *PLCG1* | *SH2D1A* | *TSC2* |
| *BAP1* | *CIC* | *ETV1* | *HRAS* | *MAGEA1* | *NF1* | *PLEKHG5* | *SMAD2* | *TSHR* |
| *BCL10* | *CKS1B* | *ETV4* | *HSP90AA1* | *MAGI1* | *NF2* | *PML* | *SMAD4* | *UBR5* |
| *BCL11A* | *CMPK1* | *EXT1* | *HSP90AB1* | *MALT1* | *NFE2L2* | *PMS1* | *SMARCA4* | *UGT1A1* |
| *BCL11B* | *COL1A1* | *EXT2* | *ICK* | *MAML2* | *NFKB1* | *PMS2* | *SMARCB1* | *USP9X* |
| *BCL2* | *CRBN* | *EZH2* | *IDH1* | *MAP2K1* | *NFKB2* | *POT1* | *SMO* | *VHL* |
| *BCL2L1* | *CREB1* | *FAM123B* | *IDH2* | *MAP2K2* | *NIN* | *POU5F1* | *SMUG1* | *WAS* |
| *BCL2L2* | *CREBBP* | *FANCA* | *IGF1R* | *MAP2K4* | *NKX2-1* | *PPARG* | *SOCS1* | *WHSC1* |
| *BCL3* | *CRKL* | *FANCC* | *IGF2* | *MAP3K7* | *NLRP1* | *PPP2R1A* | *SOX11* | *WRN* |
| *BCL6* | *CRTC1* | *FANCD2* | *IGF2R* | *MAPK1* | *NOTCH1* | *PRDM1* | *SOX2* | *WT1* |
| *BCL9* | *CSF1R* | *FANCF* | *IKBKB* | *MAPK8* | *NOTCH2* | *PRKAR1A* | *SRC* | *XPA* |
| *BCR* | *CSMD3* | *FANCG* | *IKBKE* | *MARK1* | *NOTCH4* | *PRKDC* | *SSX1* | *XPC* |
| *BIRC2* | *CTNNA1* | *FAS* | *IKZF1* | *MARK4* | *NPM1* | *PSIP1* | *STK11* | *XPO1* |
| *BIRC3* | *CTNNB1* | *FBXW7* | *IL2* | *MBD1* | *NRAS* | *PTCH1* | *STK36* | *XRCC2* |
| *BIRC5* | *CYLD* | *FGFR1* | *IL21R* | *MCL1* | *NSD1* | *PTEN* | *SUFU* | *ZNF384* |
| *BLM* | *CYP2C19* | *FGFR2* | *IL6ST* | *MDM2* | *NTRK1* | *PTGS2* | *SYK* | *ZNF521* |
| *BLNK* | *CYP2D6* | *FGFR3* | *IL7R* | *MDM4* | *NTRK3* | *PTPN11* | *SYNE1* |  |
| *BMPR1A* | *DAXX* | *FGFR4* | *ING4* | *MEN1* | *NUMA1* | *PTPRD* | *TAF1* |
| *BRAF* | *DCC* | *FH* | *IRF4* | *MET* | *NUP214* | *PTPRT* | *TAF1L* |
| *BRD3* | *DDB2* | *FLCN* | *IRS2* | *MITF* | *NUP98* | *RAD50* | *TAL1* |
| *BRIP1* | *DDIT3* | *FLI1* | *ITGA10* | *MLH1* | *PAK3* | *RAF1* | *TBX22* |
| Comprehensive Cancer Panel (Thermo Fisher Scientific : 4477685) | | | | | | | | |

| Supplementary Table 3. Mutations from COSMIC data base. | | | | |  |  |
| --- | --- | --- | --- | --- | --- | --- |
|  |  | Percentage of cases with mutation in COSMIC data base | | | | |
| Chromosome | Gene | Neuroendocrine tumor (n=353) | Solid pseudopapillary tumor (n=156) | Ductal carcinoma (n=6012) | Acinar cell carcinoma (n=83) | Pancreatoblastoma (n=23) |
| chr5 | *APC* | 3.25 | 9.09 | 3.26 | 6.14 | 0 |
| chr7 | *GRM8* | 2.08 | 0 | 26.77 | 2.38 | 0 |
| chr13 | *LAMP1* | 0.52 | 0 | 0.64 | 0 | 0 |
| chr7 | *AKAP9* | 5.73 | 0 | 5.40 | 0 | 0 |
| chr6 | *ROS1* | 5.97 | 0 | 3.87 | 3.57 | 0 |
| chr5 | *SDHA* | 0.01 | 0 | 1.78 | 0 | 0 |
| chr7 | *KMT2C* | 11.19 | 0 | 8.56 | 7.14 | 0 |
| chr1 | *DDR2* | 6.34 | 0 | 3.98 | 0 | 0 |
| chr3 | *FANCD2* | 2.08 | 0 | 1.99 | 2.38 | 0 |
| chr10 | *FGFR2* | 3.19 | 0 | 2.32 | 1.79 | 0 |
| chr, choromosome; N.D., not detected. COSMIC, v91, released Apr 7, 2020. | | | | | |  |

| Supplementary Table 4. Beta-catenin/WNT abnormalities in pancreatoblastoma | | | | |  |
| --- | --- | --- | --- | --- | --- |
| Case | Nuclear beta-catenin expressiopn | *CTNNB1* mutation | *APC* mutation | | Protein |
| Present | 1/1 | 0/1 | 1/1 | c.1816_1817insA | p.Ile606AsnfsTer28* |
| Yamaguchi et al. 2018 | 1/1 | 0/1 | 1/1 | c.5503A>G | p.R1835G** |
| Ismale et al. 2012 | 1/1 | 1/1 | 0/0 | - |  |
| Tanaka et al. 2003 | 7/7 | 2/5 | 0/3 | - |  |
| Abraham et al. 2001 | 7/9 | 5/9 | 1/3 | c.1309_1311del |  |
| Total | 17/19 (89%) | 8/17 (47%) | 3/8 (38%) |  |  |
| *Armadillo repeats; **beta-catenin binding site, SAMP repeats (Axin binding site) | | | | | |
